# Prebiotic Potential and Anti-Inflammatory Activity of Soluble Polysaccharides Obtained from Soybean Residue

**DOI:** 10.3390/foods9121808

**Published:** 2020-12-06

**Authors:** Bao Le, Thi Ngoc Anh Pham, Seung Hwan Yang

**Affiliations:** 1 Faculty of Pharmacy, Ton Duc Thang University, Ho Chi Minh City 700000, Vietnam; lebao@tdtu.edu.vn; 2 Department of Biotechnology, Chonnam National University, Yeosu 59626, Korea; 197621@jnu.ac.kr

**Keywords:** fermentation, inflammation, microwave-assisted enzymatic extraction, nutraceutical, polysaccharide, prebiotics, probiotics, soybean residue

## Abstract

In the present study, we assessed the extraction of low molecular weight soluble polysaccharides (MESP) from soybean by-products using microwave-assisted enzymatic technology and proposed the chemical structure of MESP using Fourier transform-infrared spectroscopy, gas chromatography, and ^1^H and ^13^C nuclear magnetic resonance spectrum analysis. The results suggested that MESP mainly comprised arabinose, rhamnose, and glucuronic acid with (1→4) glycosidic linkages in the backbone. Compared with inulin, MESP was found to selectively stimulate the growth of *Lactobacillus* probiotics. Moreover, the results of in vitro fermentation indicated that MESP significantly increased the concentrations of both acetate and butyrate (*p* < 0.05). MESP were treated on lipopolysaccharide (LPS)-stimulated RAW264.7 cells to determine the anti-inflammatory effect in vitro. It was observed that MESP inhibited nitric oxide, tumor necrosis factor (TNF)-α, interleukin (IL)-1β, IL-6, and IL-10 production. Furthermore, Western blotting results indicated that MESP significantly attenuated LPS-induced downregulation of phosphorylation levels of Janus kinase 2 (JAK2) and signal transducer and activator of transcription 3 (STAT3) in macrophages. The underlying mechanism might involve inhibition of the expression of pro-inflammatory cytokines, presumably via JAK2/STAT3 pathway. Collectively, the results of our study paved way for the production of MESP, which may be potentially used as nutraceutical ingredients for prebiotics and anti-inflammatory agents, from soybean residue.

## 1. Introduction

Soy *(Glycine max*) is an economical crop that has been cultivated in eastern Asian countries since ancient times, and more recently, is increasingly used in the Western countries [1]. Soybean is used for human consumption, since it is an abundant source of protein and oil. Soymilk consumption has increased in most countries; however, increased soymilk production has been continuously accompanied by soybean by-product accumulation. The residue left from soybean after aqueous extraction from soymilk production is rich in insoluble fiber (55%) and remaining protein (30%) [2]. Several studies have been conducted on soybean by-products to identify different methodologies to minimize the economic and environmental load of food production.

For decades, research has revealed that polysaccharides possess immense health beneficial physiological effects including immunomodulatory [3], antiviral [4], and antioxidant activities [5]. Soybean polysaccharide and its derivatives were reported to possess hypolipemic, hypocholesterolemic, anti-inflammatory, antidiabetic, antiobesity, anticardiovascular disease activities, and these derivatives improved metabolic syndrome. These derivatives may be related to polysaccharide activity [6,7,8]; however, soybean residue is still considered a waste product due to its low water solubility. Moreover, the correlation between the molecular weight and health benefits of polysaccharides has already been reported [7].

A decrease in the molecular weight of honey-processed *Astragalus* polysaccharide (dextran) was accompanied by its reduced anti-inflammatory activity [9]. A similar trend was observed on investigating a polysaccharide obtained from *Schizophyllum commune* [10]. He et al. [11] observed that low molecular weight polysaccharide might reach more distal regions of the colon intact and may lead to slower fermentation rate, which results in high prebiotic potency. Therefore, it is necessary to find alternative strategies to obtain low molecular weight polysaccharides with high solubility and anti-inflammatory activity from soybean residue.

Several modification methods, such as acid hydrolysis [12], autoclaving [13], and enzymatic hydrolysis [14], have been developed to prepare soluble polysaccharides. Enzymatic hydrolysis used for production of valuable polysaccharides has been intensively studied due to the improved extraction yield of water-soluble polysaccharides and the simple process; however, this method requires strict temperature and pH conditions and is time consuming [15]. In contrast, in our previous study, microwave-assisted extraction (MAE) required shorter time, reduced solvent consumption, and lower energy input [16]. Nevertheless, the effects of the combined use of enzymatic hydrolysis and MAE on the bioactivities of soybean-derived soluble polysaccharides, particularly those with low molecular weight, have not yet been investigated.

Hence, we evaluated the physicochemical characteristics and bioactivities of low molecular weight soluble polysaccharides (MESP) derived from soybean using microwave assisted enzymatic extraction (MAEE). MESP were isolated and purified via a Cellulose DEAE-52 column chromatography. The in vitro chemical characteristics and biological activities, including prebiotic and anti-inflammatory effects, of MESP were investigated.

## 2. Materials and Methods

### 2.1. Materials

The solid soy residue obtained from CJ Food Systems (Guro, Seoul, Korea) and was stored in a freezer at −4 °C before use. Inulin from chicory, lipopolysaccharide (LPS) from *Escherichia coli* O111:B4, pectinase from *Aspergillus aculeatus*, cellulase from *A. niger* were provided by Sigma Chemical (St. Louis, MO, USA). MRS carbohydrate-free broth (MB-M0757) and MRS broth (MB-M1025) were purchased from MB cell (Los Angeles, CA, USA). *Lactobacillus rhamnosus* GG ATCC 53103, *L. plantarum* ATCC 8014, *L. brevis* ATCC 367, and murine macrophage cell line RAW 264.7 ATCC TIB71 were purchased from the American Type Culture Collection. Penicillin-Streptomycin (10,000 U/mL), Dulbecco’s modified eagle’s medium, fetal bovine serum (FBS) was purchased from Gibco BRL (Grand Island, NY, USA). Bicinchoninic acid assay kit (23225) was purchased from Thermo Scientific^TM^ (Waltham, MA, USA). Griess reagent kit (G7921), tumor necrosis factor (TNF-α) (A43658), IL-6 (P26892), IL-10 (P18893), and IL-1β (P10749) were purchase from Invitrogen (Carlsbad, CA, USA). Mouse anti-β-actin (sc-47778), Janus kinase 2 (JAK2) (sc-390539), phosphorylated (p)-JAK2 (sc-101718), signal transducer and activator of transcription 3 (STAT3) (sc-8019), and p-STAT3 (sc-8059) antibodies were purchased from Santa Cruz Biotechonlogy (Santa Cruz, CA, USA). Anti-rabbit horseradish peroxidase-conjugated secondary antibodies (ab6721) was acquired from Abcam (Cambridge, MA, USA). All chemicals and reagents applied were of analytical grade.

### 2.2. Microwave Assisted Enzymatic Extraction

The fresh soybean residue was dried at 60 °C for 3 d and followed by grounding (80 mesh) in a FM-909 T mechanical grinder (Hanil Electric Co., Seoul, Korea) to obtain a homogeneous soybean residue powder. The powder was pretreated with 80% ethanol at 60 °C for 5 h to remove oligosaccharide, colored materials, and some small molecule materials. The insoluble parts were separated via centrifugation and dried until its weight was constant. The microwave extraction was performed as previously described by Le, Golokhvast, Yang, and Sun [16] with minor modifications. The pretreated powder of 10 g was accurately weighed and was immersed in 200 mL deionized water (D_2_O) (1:10 *w*/*v*) for 1 h in a 250 mL flat-bottomed flask. The mixture was hydrolyzed for 30 min using a cocktail enzyme (pectinase: cellulase = 1:1, mixed enzymes: powder = 2.0%, *w*/*v*) under the microwave irradiation power of 700 W. Thereafter, the suspension was centrifuged and concentrated to reach 20 mL by rotary evaporation under reduced pressure. Next, the mixture was precipitated with 80% (*v*/*v*) ethanol) at 4 °C overnight. The crude polysaccharide was obtained via centrifugation, deproteinized by the Sevag reagent (chloroform: n-butyl alcohol = 4:1, *v*/*v*) to remove the associated proteins, and finally lyophilized through freeze drying process. The crude polysaccharide was redissolved in distilled water and fractionated through an activated Cellulose DEAE-52 (5.0 × 30 cm) column pre-equilibrated with distilled water and was then stepwise eluted with distilled water and 0.5 M NaCl at a flow rate of 1.0 mL/min (10 mL/tube). Each fraction was evaluated at 490 nm by phenol sulfuric acid to detect the compounds. The main fraction was extensively dialyzed against ultrapure water at 4 °C for 48 h (molecular weight cut-off: 3.5 kDa), and lyophilized to obtain white pure polysaccharide, namely MESP, for further analyses.

### 2.3. Characterization of MESP

Total polysaccharide content was measure by the phenol-sulfuric acid colorimetric method at 621 nm using D-glucose as a standard. The molecular weight of MESP was measured according to a method described by Zheng et al. [17]. Monosaccharide composition of MESP was determined via a gas chromatography-mass spectrometer (6890/5973N-GC/MSD, Agilent Technologies, Santa Clara, CA, USA) after hydrolysis with 3 M trifluoroacetic acid at 100 °C for 3 h, and then the MESP were passed through a 0.45 μm syringe filter before loading on a capillary column HP-5 ms (30 m × 0.25 mm × 0.25 μm) at 25 °C. VERTEX 70 v Fourier transform infrared spectrometer (Bruker, Germany) was used for FT-IR measurements. A potassium bromide disc that contains 1% of MESP was recorded in the frequency range of 4000–400 cm^‑1^. MESP (30 mg) was maintained over P_2_O_5_ under vacuum for several days and then dissolved in D_2_O. The solid-state ^1^H and ^13^C nuclear magnetic resonance (NMR) spectra were recorded at 25 °C on a Varian Inova 500 and 600 MHz spectrometers (Varian, Walnut Creek, CA, USA), which were operated at 400 MHz and 101 MHz for ^1^H and ^13^C NMR, respectively.

### 2.4. Stimulation of Probiotic Growth

The prebiotic activity of MESP was evaluated by a method described in a previous study with minor modifications [18]. MRS carbohydrate-free broth (MB cell, Los Angeles, CA, USA) was used as the basal medium, and MESP and inulin (positive prebiotic control) were used as the individual carbon sources. Three *Lactobacillus* strains, including *L. rhamnosus* GG, *L. plantarum*, and *L. brevis*, were precultured in MRS broth, centrifuged and diluted in basal MRS medium to attain 1 × 10^7^ colony forming units (CFU)/mL. The MRS carbohydrate-free broth containing 1% carbon source (MESP or inulin) was dispensed as 200 µL aliquots into in 96-well U-shaped-bottom microplate; thereafter, 20 µL of logarithmic culture of each probiotic strains was added for fermentation. The growth of the probiotics was evaluated after 24 h of incubation using an ELISA reader (BioTek Instruments, Winooski, VT, USA) at a wavelength of 600 nm.

### 2.5. Short Chain Fatty Acid (SCFA) Analysis

In vitro fermentation was performed according to a previously described method [19] with certain modifications. Fresh feces samples were obtained from three healthy individuals (2 female, 1 male, aged 22–29 years), who had not been treated with antibiotics in the last 3 months. Feces were homogenized with 0.1 M anaerobic phosphate-buffered saline (pH 7.0) using a Bead Ruptor Homogenizer (Omni International, NW Kennesaw, GA, USA) to make 10% (*w*/*v*) slurries. The basic medium comprised the followings components (per liter): 0.45 g KH_2_PO_4_; 0.45 g K_2_HPO_4_; 0.05 g NaCl; 0.064 g KCl; 1.0 g MgSO_4_·7H_2_O; 0.5 g cysteine-HCl; 0.5 g bile salts, 0.05 g hemin; 0.001 g resazurin; 2.0 mL Tween 80; 0.01 mL vitamin K, with or without supplement of 8.0 g each of MESP or inulin. The fermentation was carried out in an anaerobic chamber at 37 °C for 24 h. After fermentation, the culture (1 mL) was dispensed into a sterile Eppendorf tube (1.5 mL) and centrifuged at 13,000× *g* for 10 min to collect the supernatant. Next, the supernatants were filtered using organic phase microfiltration membrane (0.22 mm) and analyzed by Agilent 1100 Series (Agilent Technologies, Inc., Santa Clara, CA, USA) with a DAD detector at 210 nm equipped with a calcium-loaded Aminex-HPX-87C column (Bio-Rad Corp., Richmond, CA, USA).

### 2.6. Cytotoxicity Assay

We examined the cytotoxicity of MESP on RAW 264.7 murine macrophage cells by growing them in Dulbecco’s modified eagle’s medium supplemented with combination of penicillin and streptomycin (final concentration of 100 units/mL and 100 µg/mL, respectively), along with 10% fetal bovine serum at 37 °C, with 5% CO_2_ supply. As the cells reached 80% confluence, they were treated with different concentrations of MESP (5–1280 μg/mL) for 24 h. The cell vitality was measured using methylthiazolyldiphenyl-tetrazolium bromide (MTT) assay using microplate reader (F50, Tecan, Mannedorf, Switzerland) via spectrophotometry at 570 nm.

### 2.7. Measurement of NO and Cytokines

To evaluate anti-inflammatory potential of MESP, RAW264.7 cells (5 × 10^5^ cells/mL) were seeded in a 24-well plate and adding MESP at various concentrations in the presence of LPS (1 µg/mL) in 5% CO_2_ at 37 °C for 24 h. The NO, TNF-α, IL-6, IL-10, and IL-1β concentrations in the supernatant were measured by Griess and ELISA assays according to the manufacturer’s instructions.

### 2.8. Western Blot Analysis

RAW264.7 cells were rinsed with cold 1× phosphate buffer saline and detached with EDTA-free 0.25% trypsin, followed by homogenization using RIPA lysis buffer (Sigma-Aldrich, St. Louis, MO, USA). After quantitative determination of the protein content by BCA assay kit, equal quantities of total proteins were separated by denaturing them via 10% SDS-PAGE and then transferred to PVDF membranes (GE Healthcare, Buckinghamshire, UK). The membranes were then incubated in Tris-buffered saline containing 0.1% (*v*/*v*) Tween-20 (TBST) and 5% skim milk at 37 °C for 3 h, and the specific primary antibodies were incubated at 4 °C overnight. After several washes with TBST (0.075%), they were incubated with anti-rabbit horseradish peroxidase-conjugated secondary antibodies for 1 h at room temperature. The targeted protein blots were developed using an enhanced chemiluminescence (ECL) kit (ECL-plus, Thermo Scientific, USA) and detected by using a Chemiluminometer (CLINX Scientific Instrument Co., Ltd., Shanghai, China).

### 2.9. Statistical Analysis

All results were performed in triplicate and expressed as mean ± standard deviation. Analysis of variance and Student’s t-test were analyzed using the SPSS software (IBM, SPSS 22.0, Chicago, IL, USA) at *p* < 0.05 and *p* < 0.01.

## 3. Results

### 3.1. Polysaccharide Extraction, Isolation, and Purification

**Figure 1:**
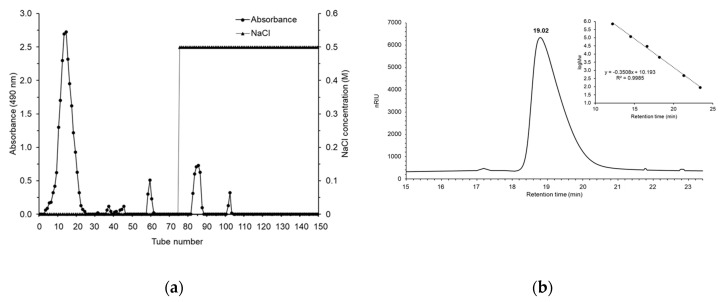
Elution curve obtained using Sephadex G-75 gel filtration (**a**) and HPLC chromatograms (**b**).

Four fractions were obtained by NaCl (0 and 0.5 M) elution (Figure 1a). Each polysaccharide peak was collected, dialyzed, and lyophilized. Due to the lower yields observed for other eluted fractions (1.72–11.41%), the main MESP fraction (80.86%) was selected for structural and functional characterizations. The single and symmetrically sharp peak at 19.02 min in the MESP profile indicated that MESP was a homogeneous polysaccharide with molecular weight of 3.32 kDa, based on the calibration curve of dextran (Log M_w_ = −0.3508 × retention time + 10.193; Figure 1b). The total sugar content of MESP was 97.69% with remaining trace amounts of protein and nucleic acid.

Gas chromatography (GC) analysis of the monosaccharides (Table 1) revealed that MESP comprised arabinose, galacturonic acid, rhamnose, xylose, galactose, glucose, and fucose at a molar ratio of 27.4:18.1:11.41:5.71:2.53:1.53:1.32, indicating that arabinose, galacturonic acid, and rhamnose were the main monosaccharide components of low molecular weight soluble polysaccharides (MESP).

### 3.2. Structural Characterization

**Figure 2:**
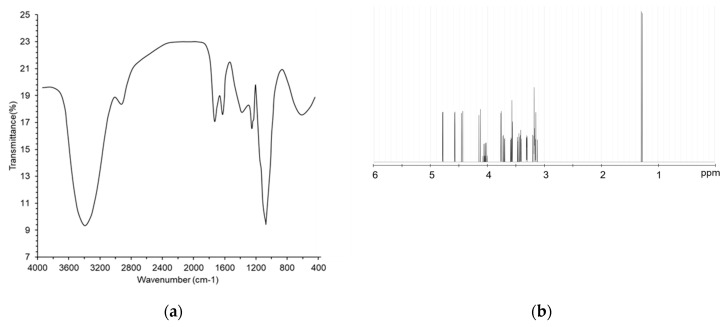

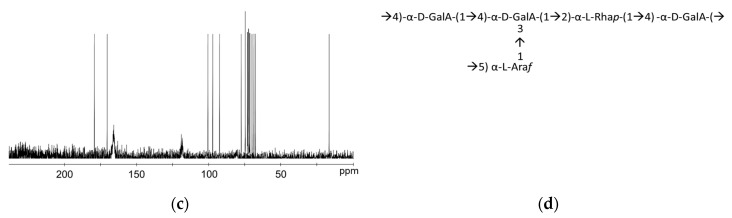
Fourier transform infrared spectra (**a**); ^1^H-NMR spectra (**b**); ^13^C-NMR spectra (**c**); and proposed structure (**d**) of low molecular weight soluble polysaccharides (MESP).

The integrated structure of MESP was further assessed via FT-IR and ^1^H and ^13^C NMR. The typical FT-IR spectrum of MESP in the 400–4000 cm^−1^ range is presented in Figure 2a. The graft MESP revealed strong stretching peak at 3397.8 cm^−1^ corresponding to the hydroxyl groups (O-H) [20]. The weak absorption peak at 2919.2 cm^−1^ was characteristic of C-H asymmetric stretching vibration [21]. The bands at 1736.2 and 1629.6 cm^−1^ might be due to the nonsymmetrical vibration of C=O in the ester group [22]. Moreover, the absorption signals at around 1473.3 and 1074.1 cm^−1^ indicated the presence of residual water (OH^-^). Furthermore, the signal observed at 1256.3 cm^−1^ was attributed to the stretching vibration of C-O. Collectively, FT-IR analysis suggested that MESP possessed typical sugar groups.

The ^1^H and ^13^C NMR spectra, illustrated in Figure 2b,c, confirmed the attribution and structural characterization of MESP. Based on the data in existing literature, the signal of five anomeric protons at δ3.18–4.18 was identified as the zone of accumulation of protons from α-D-GalA^-^ residues, whereas that at δ1.21–4.03 was assigned to α-L-Rha*p*^-^ [8]. The ^13^C NMR spectrum of MESP showed no signal at low field from 160 to 180 ppm, indicating that MESP did not contain uronic acid. Chemical shifts of MESP are summarized in Table 2. According to the corresponding chemical shifts in existing literature, MESP structure was proposed, as shown in Figure 2d.

### 3.3. MESP Stimulated Probiotic Growth

**Figure 3:**
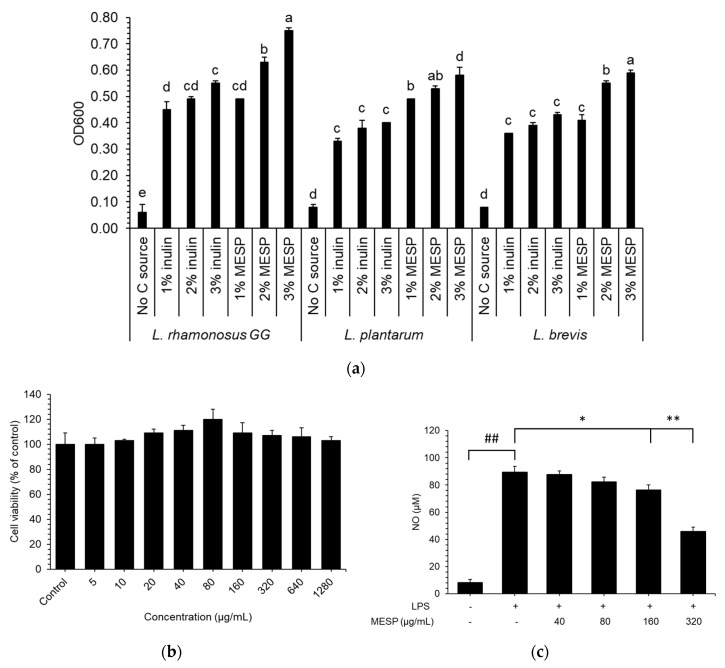
Growth of probiotics in glucose free-MRS base media with supplement of inulin or MESP (**a**). OD600, optical density of a sample measured at a wavelength of 600 nm. Values are expressed as mean ± SD (*n* = 3). Different letters indicate significant (*p* < 0.05) differences in the same strain. Cytotoxic effects (**b**) and NO production (**c**) of MESP in RAW 264.7 cells. ## *p* < 0.01, compared to the control group; * *p* < 0.05, ** *p* < 0.01, compared to the lipopolysaccharide (LPS) treated group.

The number of all tested *Lactobacillus* strains treated with MESP or inulin was remarkably increased (Figure 3a), indicating that all *Lactobacillus* strains could utilize MESP and inulin. *Lactobacillus* exhibited an outstanding growth rate when MESP, in contrast to inulin, was used as the carbon source.

### 3.4. Effect of MESP on SCFA Production

The pH of the cecum is lower than that of the ileum, which in turn affects the growth inhibition of pH-sensitive pathogenic bacteria, thereby altering gut microbiota composition and promoting host health. After in vitro fermentation, the concentrations of total short chain fatty acids (SCFAs), including acetic, butyric, and propionic acids, were significantly higher (*p* < 0.05) in the MESP group than those in the control group (Table 3), indicating that MESP enhanced SCFA production in the gut model. A comparison of the results revealed that the concentrations of total SCFAs were significantly higher (*p* < 0.05) after MESP treatment than those after inulin treatment. Acetic acid concentration was significantly increased (*p* < 0.05).

### 3.5. Effects of MESP on RAW264.7 Cell Proliferation

We analyzed the safe concentration of MESP on RAW 264.7 macrophage, with various MESP concentrations wherein cell viability was determined by MTT assay. We observed that MESP at concentrations 5–1280 μg/mL did not affect cell growth in a dose-dependent manner. (Figure 3b).

### 3.6. Effects of MESP on NO Production in LPS-Stimulated RAW264.7 Cells

The NO levels were markedly induced after treatment with LPS alone, whereas they were remarkably inhibited by MESP pretreatment in a dose-dependent manner (Figure 3c). As 160 μg/mL MESP did not present any cytotoxic effect, we propose the possibility that inhibition of NO production was not linked to the cytotoxic effects on RAW264.7 cells.

### 3.7. Effect of MESP on IL-6, TNF-α, and IL-1β Release in LPS-Stimulated RAW264.7 Cells

**Figure 4:**
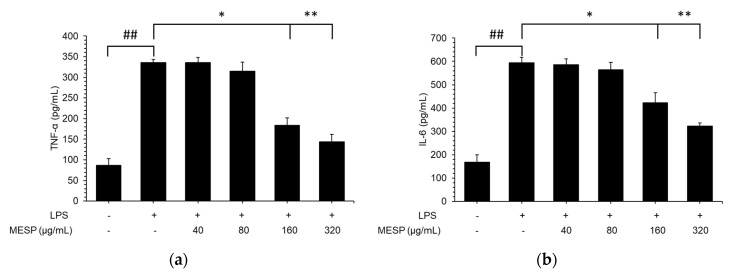

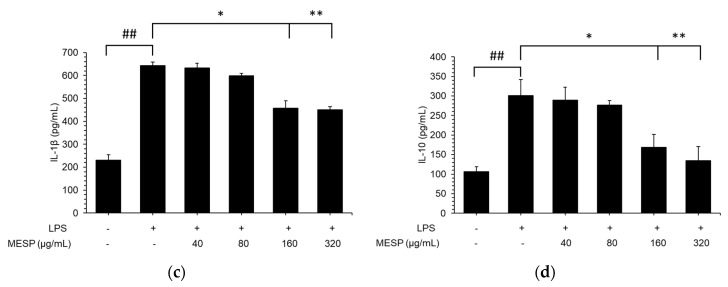
Inhibitory effects of MESP on TNF-α (**a**), IL-6 (**b**), IL-1β (**c**), IL-10 (**d**) production on RAW 264.7 cells by ELISA. Values are expressed as mean ± SD (*n* = 3). ## *p* < 0.01, compared to the control group; * *p* < 0.05, ** *p* < 0.01, compared to the LPS treated group.

As illustrated in Figure 4, inflammatory cytokine production was tremendously increased in the LPS-treated group compared with that in the control group (*p* < 0.01), whereas TNF-α, IL-6, IL-1β, and IL-10 production was significantly inhibited by the six tested peptides in LPS-treated RAW264.7 cells, compared with that in the control cells. Treatment with MESP at concentration 20 µg/mL showed the strongest inhibitory activity towards TNF-α, IL-6, IL-1β, and IL-10 at 57.2%, 30.1%, 45.7%, and 55.3%, respectively.

### 3.8. MESP Blocked the LPS-Triggered Inflammatory Response via the JAK2/STAT3 Pathway

**Figure 5:**
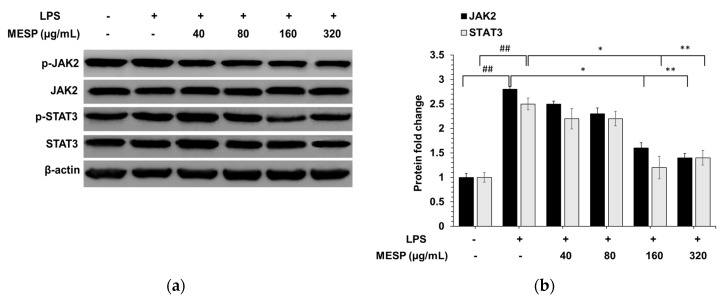
Inhibitory effects of MESP on JAK2/STAT3 pathway activation. Immunoblot analysis of JAK2 and STAT3 protein levels (**a**) and quantification of protein levels (**b**) normalized to β-actin. ## *p* < 0.01, compared to the control group; * *p* < 0.05, ** *p* < 0.01, compared to the LPS treated group.

We revealed that LPS induced JAK2 and STAT3 phosphorylation (Figure 5). Next, we explored the role of MESP in JAK2 and STAT3 regulation. As illustrated in Figure 5, MESP could significantly suppress phosphorylated (LPS-induced) JAK2/STAT3, indicating that MESP exerted anti-inflammatory effects through the JAK2/STAT3 signaling pathway.

## 4. Discussion

In the present study, crude polysaccharide was extracted from soybean residue by MAEE extraction and then fractionated using the Cellulose DEAE-Sephadex A-25 column. The extraction yield was 73.8%, which is comparable to that (previously reported value ranging from 7.09–85%) obtained using alkaline hydrogen peroxide, enzymatic hydrolysis, autoclaving, fermentation, and ultrasonic-assisted extraction [23,24,25]. In MAEE, the extraction time was shorter than that in microwave-assisted extraction (0.5–2 h), whereas the extraction temperature was lower than that for autoclaving (121 °C); moreover, enzyme loading of MAEE extraction was lower than that of enzymatic hydrolysis using cellulase and pectinases, thereby suggesting that MAEE extraction was more efficient and eco-friendly. In a previous study, numerous heterogeneous polysaccharides isolated from soybean residue (okara) presented monosaccharide compositions comprising mainly galacturonic acid, galactose, arabinose, xylose, fucose, and rhamnose [2,26]. Furthermore, enzyme treatment increased arabinose content in polysaccharides obtained from tea leaf and pulp [27,28], and in accordance with our results. This demonstrated that MESP was a pectin-like polysaccharide with rhamnose and galacturonic acid forming its backbone.

To evaluate the growth stimulating effects of MESP on probiotics, MESP was used as the single carbon source for *Lactobacillus* spp. cultivation. Meanwhile, the medium without a carbon source was used as a negative control, whereas inulin (commercial prebiotic) was used as a positive control to compare its effects with those of MESP. Our results were in accordance to those of previous in vitro and in vivo studies, which revealed that soybean oligosaccharides and soluble polysaccharides could stimulate *Lactobacillus* and *Bifidobacterium* growth [29,30,31]. Soybean soluble polysaccharides, which are rich in GalA and Ara, were reported to stimulate *Lactobacillus fermentum* and *L. plantarum* growth by 49.5 and 5.25 times, respectively [29]. A similar result was observed, demonstrating that consumption of high-dose soybean oligosaccharides could promote *Bifidobacterium* and *Lactobacillus* proliferation in a mouse model [32]. This indicated that MESP was a good substrate for facilitating probiotic growth.

SCFAs are the main end-products obtained fermentation of dietary fiber by gut microbes in the colons [19]. This increase was consistent with the change in the number of *Lactobacillus* spp. (known as lactate producers). Enriched acetate production has been reported in numerous inulin intervention studies [14,29]. Moreover, a similar result has been documented when soybean oligosaccharides were used. Zhou, et al. [33] found that the fermentation of mini-pig-supplemented soybean oligosaccharides increased acetic, propionic, and butyrate acid production, and this could be corrected by increasing the number of *Lactobacillus* spp. Our results indicated that MESP exerted better effects on SCFA production and richness than inulin, and this might be due to the more diverse monosaccharide compositions of MESP.

Macrophages are crucial immunocytes and play a pivotal role in protecting the host from certain pathogens and maintaining homeostasis [34]. Our result revealed that MESP exerted no toxicity toward RAW264.7 cells, and this was in accordance with the results of previous studies [32,35]. Moreover, soybean oligosaccharide was considered “generally recognized as safe” material in the United States. To estimate the suppressive effects on NO, pro-inflammatory mediator in LPS-induced inflammation, RAW264.7 cells were treated with MESP at serial concentrations for 4 h before treatment with LPS (1 µg/mL). These data suggested that MESP exerts anti-inflammatory activity towards RAW264.7 cells. To determine the underlying anti-inflammatory mechanisms of MESP, we evaluated the production of inflammatory cytokines, which are crucially involved in the inflammatory response. The immunomodulatory capacities were affected by the different monosaccharide compositions of the MESP, and this was in accordance with the results of a previous study [35]. Furthermore, the (1→4) glycosidic bond, which is the main type of linkage formed in MESP, might partly be responsible for the immunoregulatory activity of MESP [36,37]. A previous study showed that soybean curd residue at higher concentrations (800–1600 µg/mL) significantly downregulated LPS-induced TNF-α and IL-1β protein expression [38]. Additionally, admixtures of soybean soluble polysaccharides and genistein reduced IL-1, IL-6, and TNF-α levels in high-dose L-carnitine-fed mice [39]. Recent studies have confirmed the importance of JAK2/STAT3 in the macrophage inflammatory response [40,41]. STAT3 is a protein that mediates the expression of numerous cytokines such as IL-6 to promote inflammation. p-STAT3 can subsequently translocate into the nucleus and bind to DNA response elements, and it is generally activated by regulating the activity of the cytoplasmic tyrosine kinase JAK2 [42]. Further evidence that the JAK2/STAT3 signaling pathway could be activated by a series of cytokines, including TNF-α, IL-1β, and IL-6, has been presented [43].

## 5. Conclusions

The present study revealed that the use of MAE extraction, followed by anion exchange chromatography, allowed the production of low molecular weight (3.32 kDa) MESP from soybean residue. MESP mainly comprised galacturonic acid, rhamnose, and arabinose with copious (1→4) glycosidic linkages in its backbone. MESP exhibited remarkable in vitro prebiotic properties, including stimulation of *Lactobacillus* growth and SCFA production. Furthermore, MESP possessed potential anti-inflammatory activity that might be mediated by inhibiting NO, IL-6, IL-1β, IL-10, and TNF-α production by suppressing the JAK2/STAT3 pathway in LPS-stimulated RAW264.7 cells.

## Figures and Tables

**Table 1:** Monosaccharide composition of okara polysaccharide.

Sample	Molecular Weight (kDa)	Monosaccharide Compositions (mol%)							
		Ara	Rha	Fuc	Xyl	Man	Gal	Glc	GalA
Soybean residue		16.41	3.46	0.91	12.89	ND	27.56	10.23	20.3
MESP	3.4	27.4	11.41	1.32	5.71	ND	2.53	1.53	18.1

**Table 2:** Summary of ^1^H and ^13^C chemical shifts for MESP.

Residues		Chemical Shift Assignment of MESP						
			1	2	3	4	5	6
A	→4)-α-D-GalA-(1→	H	4.58	3.18	3.58	3.42	3.76	
		C	94.3	74.0	74.9	73.2	76.4	172.2
B	→2)-α-L-Rha*p*-(1→	H	4.53	3.57	3.46	3.15	4.03	1.21
		C	99.0	79.2	69.4	71.7	70.4	18.3
C	α-D-GalA-(1→	H	4.45	3.18	3.33	3.71	4.14	
		C	98.4	74.1	74.2	73.5	74.6	181.1
D	→5) α-L-Ara*f* (1→	H	4.88	3.56	3.52	3.37	3.95	
		C	98.59	72.2	69.6	71.23	61.01	15.9

**Table 3:** Concentrations of short chain fatty acids (SCFAs) at different points during in vitro fermentation.

SCFAs (mM)	Sample	Fermentation Time (h)				
		0	6	12	24	48
Acetic acid	Control	3.89 ± 0.48 H ^1^	4.29 ± 0.69 cH	16.48 ± 0.71 bF	15.39 ± 0.67 cF	15.48 ± 0.99 cF
	1% inulin		9.48 ± 0.49 bG	21.86 ± 0.89 bE	29.82 ± 0.08 bC	24.36 ± 0.53 bD
	1% MESP		24.18 ± 0.36 aD	51.84 ± 0.05 aB	69.48 ± 0.74 aA	69.14 ± 0.62 aA
Butyric acid	Control	2.18 ± 0.31 E	2.95 ± 0.12 bE	4.85 ± 0.49 bD	8.62 ± 0.18 bB	6.21 ± 0.83 cC
	1% inulin		4.31 ± 0.81 aD	6.14 ± 0.48 aC	9.89 ± 0.84 bB	9.18 ± 0.23 bB
	1% MESP		4.18 ± 0.69 aD	6.83 ± 0.41 aC	13.21 ± 0.55 aA	13.23 ± 0.81 aA
Propionic acid	Control	1.22 ± 0.23 D	1.33 ± 0.04 bD	2.89 ± 0.63 bD	4.26 ± 0.47 bC	4.11 ± 0.84 bC
	1% inulin		4.15 ± 0.28 aC	6.81 ± 0.16 aB	11.51 ± 0.04 aA	11.81 ± 0.41 aA
	1% MESP		4.92 ± 0.85 aC	7.21 ± 0.74 aB	12.21 ± 0.56 aA	12.18 ± 0.07 aA
Total SCFAs	Control	9.12 ± 0.11 G	9.34 ± 0.75 cG	22.14 ± 0.44 cE	34.95 ± 0.12 bD	32.43 ± 0.64 cD
	1% inulin		11.15 ±0.07 bF	33.81 ± 0.49 bD	45.12 ± 0.17 bC	44.26 ± 0.16 bC
	1% MESP		29.81 ± 0.09 aE	63.31 ± 0.18 aB	83.49 ± 0.78 aA	82.46 ± 0.36 aA

^1^ Values are expressed as means ± SD (*n* = 3). Values followed by the same capital letters are not significantly different at *p* < 0.05 among different samples at the same time point, while the same minuscules letter are not significantly different among different times (*p* < 0.05) in the same SCFAs.
